# Stress, Burnout, and Associated Risk Factors in Medical Students

**DOI:** 10.7759/cureus.6633

**Published:** 2020-01-12

**Authors:** Asem Shadid, Abdullah M Shadid, Abdulrahman Shadid, Faisal E Almutairi, Khalid E Almotairi, Talal Aldarwish, Omar Alzamil, Feras Alkholaiwi, Salah-Ud-Din Khan

**Affiliations:** 1 Medicine, Al Imam Mohammad Ibn Saud Islamic University, Riyadh, SAU; 2 Medicine, King Saud University, Riyadh, SAU; 3 Otorhinolaryngology - Head and Neck Surgery, Al Imam Mohammad Ibn Saud Islamic University, Riyadh, USA; 4 Biochemistry, Al Imam Mohammad Ibn Saud Islamic University, Riyadh, SAU

**Keywords:** burnout, stress, medical students, extracurricular activities, medical education, coping strategies, academic performance

## Abstract

Objectives

To determine the prevalence of and the risk factors associated with burnout and stress for medical students in Saudi Arabia.

Methods

A cross-sectional, survey-based study was distributed between January and February 2018 among all 500 medical students from the first to fifth years in a medical college; 356 of the students responded (71.2% response rate). Burnout was measured using the Maslach Burnout Inventory-Student Survey (MBI-SS) while the stress level was measured using the 12-item General Health Questionnaire (GHQ-12). Socio-demographics, professional characteristics, and participation in extracurricular activities were also included as possible predictors of burnout and stress.

Results

The study revealed that the stress level was (51.7%, n= 184) and the rate of high burnout was (38.2%, n= 136), expressing high exhaustion (77.8%, n=277), high cynicism (65.7%, n=234), and low academic efficiency (45.5%, n=162). Half of the students (50%, n=178) participated in extracurricular activities and were involved in one or more activities such as organizing activities and medical volunteering (n = 52, 14.6%), research (n = 59, 16.6%), and physical exercise (n = 71, 10.4%). There was a statistically significant positive correlation between overall burnout and a lower grade point average (GPA) (OR = 0.581, p 0.004, 95% CI = 0.400 to 0.843). A statistically significant positive correlation was found between stress and students with a lower GPA (OR = 0.737, P = 0.0.23, 95% CI = 0.566 to 0.959); stress was also higher in students who were not involved in any extracurricular activities (OR 1.893, P = 0.004, 95% CI = 1.22 to 2.918).

Conclusion

Our study shows high burnout rates among medical students. Low GPA students in this study showed a higher overall burnout. Stress was high in our study participants and was higher in students with a low GPA and in students who were not involved in any extracurricular activities.

## Introduction

Medical schools are known to be a stressful environment that often leads to a negative effect on the students' academic performance and physical and psychological health. Worldwide, medical students are expected to take responsibility, learn an endless amount of information, engage in multiple activities, and make a huge effort with limited time and energy. This challenges students to develop excellent skills, and meet requirements to be good practitioners. Thus, constant high demand can lead to burnout and stress, which may last throughout the training and beyond [1].

Burnout syndrome is considered to be a public health issue because it has a mental, social, and physical association on people in their workplaces, with a clear effect on the condition of life and performance [1-3]. The burnout concept was first described in the 1970s and referred to as a prolonged reaction to chronic emotional and interpersonal stressors on the job. Burnout is defined as a multidimensional syndrome known by the three dimensions: 1) emotional exhaustion, 2) depersonalization, and 3) a diminished feeling of personal achievement [2-3]. Burnout is a work-related matter, unlike stress, which may be experienced in all life forms [1-3]. Stress is known as any uncomfortable emotional experience accompanied by predictable biochemical, physiological, and behavioral changes [4].

Medical students use extracurricular activities, physical sports, and other different methods for coping with burnout and stress [5-8]. Studies show a lack of coping strategies may generate a high level of stress with more vulnerability to develop burnout [7-8]. Extracurricular activities, considered one of the coping mechanisms shown to have a good effect on mental and physical health, can reduce stress, anxiety, and burnout [8].

Although there are a lot of studies about the prevalence of burnout globally, it is important to have a better explanation and understanding of the factors associated with burnout and stress. In addition, there is little known about burnout and stress among medical students in Saudi Arabia. To the best of our knowledge, there are no studies that focus on the association between grade point average (GPA) and failure rate with its impact on stress and burnout levels together. Accordingly, the objective of this study was to determine: 1) the prevalence of psychological stress and burnout levels among preclinical and clinical years’ medical students at a medical school in Saudi Arabia, 2) assess the association with extracurricular activities in adapting with stress and burnout, and 3) have a better clarification and understanding of the risk factors associated with burnout and stress.

## Materials and methods

Study design and population

A cross-sectional study was conducted between January and February 2018 among medical students at Al Imam Mohammad ibn Saud Islamic University (IMSIU) in Riyadh, Saudi Arabia. All students (n=500) from preclinical years (first to third) and clinical years (fourth and fifth) were approached. The questionnaire was completed by 356 students, resulting in a 71.2% response rate. This research was approved by the Institutional Review Board of the medical research center, Al Imam Mohammad ibn Saud Islamic University, and adheres to the tenets of the Declaration of Helsinki (approval number: HAPO-01-R-010). Participation was voluntary and written informed consent was obtained before filling the questionnaire.

Survey instruments

The students were asked to complete the questionnaire consisting of three parts: (1) demographic variables such as gender, age, marital status, living arrangement, academic year, current grade point average (GPA), having ever failed any course/block in college, smoking, having a physician among first-degree family members, and participation in extracurricular activities; (2) the previously validated 12-item General Health Questionnaire (GHQ-12) [9]; and (3) previously used and validated Maslach Burnout Inventory-Student Survey (MBI-SS) [10-11].

GHQ-12 is one of the most widely used tools to measure stress levels [8-9]. It consists of 12 items, with each of them evaluating the manifestations of stress over a few weeks preceding the study. The GHQ 12 utilizes a four-point Likert scale scoring system. Participants respond to each question by choosing from four possible responses: “not at all,” “no more than usual,” “rather more than usual”, and “much more than usual.” After that, a binary scoring method is used to evaluate responses where replies are coded 0-0-1-1. This scoring system assigns a score of one to the two most symptomatic answers and a score of zero to the two least symptomatic answers so that the final score for each question takes a value of 0 or 1. Due to the high scores obtained using the GHQ-12 survey, the mean value was used as a cut-off value to indicate stress.

The MBI-SS consists of three dimensions that assess burnout: 1-emotional exhaustion, which is defined as severe fatigue caused by study demands, and it represents the basic individual stress component of the syndrome; 2-cynicism, which can be defined as the student’s mental distance from his/her studies or excessively detached responses to other students at an academic setting, representing the interpersonal component of burnout; and 3-reduced academic efficacy, which can be defined as feelings of decline in one’s competence and productivity and to a lowered sense of accomplishment, representing the self-evaluation component of burnout [3,10-11]. Emotional exhaustion was measured using five questions (low = 0-9; moderate = 10-14; high > 14), while cynicism was measured using four questions (low = 0-1; moderate = 2-6; high > 6), academic efficacy was measured using six questions (low ≤ 22; moderate = 23-27; high ≥ 28). The frequency for each item was scored on a seven-point Likert scale and ranges from 0 (Never) to 6 (Always). High scores on emotional exhaustion, cynicism, and low scores on academic efficacy are indicative of burnout (academic efficacy items are reverse scored so that low scores indicate low academic efficacy and thus a higher burnout). Elevated scores for emotional exhaustion and cynicism and low scores for academic efficacy indicate high levels of burnout. The three-dimensional criteria (high scores for emotional exhaustion and cynicism and low scores for academic efficacy) were used as the criteria for the diagnosis of burnout.

Statistical analysis

Performed using Statistical Package for Social Sciences (SPSS) version 24.0 (IBM Corp, Armonk, NY). The scores for the GHQ-12 and the MBI-SS total and subscales were calculated. Data were summarized as counts and percentages. The reliability of the survey was assessed using Cronbach's alpha with values greater than 0.7 indicating good reliability. This was performed for the GHQ-12 survey as well as each scale of the MBI-SS survey. The chi-square test was used to assess the association of various demographics, such as GPA, failing courses with high stress (score greater than mean GHQ-12 score), and high burnout as defined above. The chi-square test of independence was used due to the nature of variables that are categorical in nature. This test was used to assess whether the distribution of high burnout and stress was significantly different from what is expected under the null hypothesis. The chi-square test for linear trend was used when the independent variable was ordinal in nature (academic year and GPS) to assess whether any significant trend exists.

Logistic regression was then performed to assess independent predictors of stress and high burnout. The regression model included variables with a p-value of less than 0.1 in the initial analysis. GPA was included as a continuous predictor to assess the trend of association rather than the association of each GPA category with burnout. Forward and backward approaches were used while constructing the models to assess the model which best fits the data. Two-tailed hypothesis testing was performed. P-values less than 0.05 were considered statistically significant. Demographic variables and extracurricular activities as the independent variables. Odds ratios (OR) and 95% confidence intervals (95% CI) were calculated. A p-value of less than 0.05 was considered statistically significant throughout the analysis.

## Results

The questionnaire was completed by 356 students. Males represented n = 224 (62.9%) while females represented n = 132 (37.1%). Most of the students were aged 18 to 24 years (n = 332, 93.3%) while the remaining students were older than 25 years (n = 24, 6.7%). Most of the students were single (n = 343, 96.3%) and most of them were living with their parents or relatives (n= 334, 93.8%). The sample collected included students from academic years one through five as, thus, all academic years were well-represented (Table 1).

**Table 1:** Demographic characteristics of the study participants (n = 356) GPA: grade point average

		n (%)
Gender	Female	132 (37.1)
	Male	224 (62.9)
Age	18 – 24	332 (93.3)
	>25	24 (6.7)
Marital status	Single	343 (96.3)
	Married	13 (3.7)
Living arrangement	Alone	11 (3.1)
	Other	2 (0.6)
	Outside the dorms	2 (0.6)
	Parents/relatives	334 (93.8)
	Student dorms	7 (2)
Academic year	1st year	83 (23.3)
	2nd year	103 (28.9)
	3rd year	72 (20.2)
	4th year	53 (14.9)
	5th year	45 (12.6)
GPA	2 - 2.74	14 (3.9)
	2.75 - 3.74	87 (24.4)
	3.75 - 4.49	153 (43)
	4.50 - 5	102 (28.7)
Ever failed any course/block in college?	No	256 (71.9)
	Yes	100 (28.1)
Smoker	No	278 (78.1)
	Yes	78 (21.9)
Physician among first-degree family members	No	254 (71.3)
	Yes	102 (28.7)

Half of the medical students were not involved in any extracurricular activities (n = 181, 50.8%) while the remaining were involved in one or more activity such as organizing activities and medical volunteering (n = 52, 14.6%), research (n = 59, 16.6%), and sports (n = 71, 10.4%). See Table 2.

**Table 2:** Extracurricular activities among the study participants

	No		Yes	
	n	%	n	%
None	181	50.8%	175	49.2%
Student activity club	290	81.5%	66	18.5%
Organizing activities and medical volunteering	304	85.4%	52	14.6%
Research	297	83.4%	59	16.6%
Physical exercise	285	80.1%	71	19.9%
Video gaming	319	89.6%	37	10.4%
Other	300	84.3%	56	15.7%

Results show that 77.8% (n = 277) of the medical students included were suffering from high exhaustion while 65.7% (n = 234) suffered from high cynicism. Academic efficiency was low in 45.5% (n = 162) of the medical students. Overall burnout was present in 38.2% (n = 136) of the students. See Table 3.

**Table 3:** Burnout and stress across medical students MBI-SS: Maslach Burnout Inventory-Student Survey; GHQ-12: General Health Questionnaire

	Low		High	
	Count	Percentage	Count	Percentage
MBI-SS				
Exhaustion	79	22.2%	277	77.8%
Cynicism	122	34.3%	234	65.7%
Academic efficiency	162	45.5%	194	54.5%
Overall burnout (3 Dim)	220	61.8%	136	38.2%
GHQ-12				
Stress	172	48.3%	184	51.7%

Emotional exhaustion (EE)

Regarding the exhaustion scale in the MBI-SS survey, gender was significantly associated with high exhaustion where more females showed exhaustion as compared to males (87.9% vs. 71.9%, P < 0.001). Interestingly, GPA showed a significant association with exhaustion where exhaustion decreased with increasing GPA from 2 to 5 (92.9%, 82.8%, 77.8%, and 71.6%, respectively). Ever failing a course in college was associated with higher exhaustion (85% vs. 75%, P = 0.041). Smoking was associated with exhaustion although the association did not reach statistical significance (85.9% vs. 75.5% in smokers and non-smokers, respectively). Extracurricular activity was associated with exhaustion, medical students who did not have any extracurricular activity suffered from exhaustion to an extent greater than that seen in students who had an extracurricular activity (85.7% vs. 70.2%, P < 0.001). Research activity was also associated with exhaustion where medical students who participated in research activities suffered from exhaustion to an extent lower than that seen in medical students who did not participate in research activities (55.9% vs. 82.8%, P < 0.001). Academic year, marital status, physical exercise, and video gaming were not associated with exhaustion (P>0.05).

Cynicism (Cyn)

Academic year, GPA, and ever failing a course in college were significantly associated with high cynicism (P = 0.0066, 0.001, respectively). A higher academic year and a lower GPA were significantly associated with higher cynicism. Failing a course in college was also significantly associated with higher cynicism (78% vs. 60.9%, P = 0.002). None of the other variables showed a significant association with cynicism.

Academic efficiency (AE)

GPA was significantly associated with academic efficiency where academic efficiency increased with increasing GPA and the association was statistically significant when the chi-square test for linear trend was used (P < 0.001). High academic efficiency was more prevalent in individuals who have a first-degree relative working as a physician (58.3% vs. 45.1%, P = 0.024). None of the remaining variables was significantly associated with academic efficiency. High academic efficiency was more prevalent in medical students who did not fail any course in college (50% vs. 34%, P = 0.006).

Overall burnout

GPA was significantly associated with high burnout (P < 0.001). The proportion of individuals with high burnout was highest among individuals with GPA 2 - 2.74 and showed a constant decrease till the proportion was lowest among individuals with a GPA of 4.5 - 5. High burnout was also significantly associated with failing a course/block in college. Individuals who previously failed a course or block in college were at higher risk of high burnout compared to individuals who did not fail a course or a block before (56% vs. 31.3%, P < 0.001). Not participating in activities was also significantly associated with high burnout. Individuals who did not participate in activities suffered from burnout to an extent greater than that seen in individuals who participated in activities (43.4% vs. 33.1%, P = 0.042). Individuals who do sports suffered from burnout to an extent lower than that seen in those who did not do sports (28.2% vs. 40.7%, P = 0.052).

Stress

Various factors were associated with stress. Stress was more prevalent in students who lived with their parents or relatives as compared to those who did not (53.3% vs. 27.3%, P = 0.018). Lower GPA <3.75 (out of 5) was associated with higher stress (P = 0.01). Being involved in extracurricular activities was significantly associated with lower stress (P = 0.002). High stress was less prevalent in members of the student activities club (P = 0.027). Student activities club, are student-focused extracurricular clubs and programs at the college. It's generally designed to allow students the opportunities in leadership, social responsibility, volunteerism, and research activities. High stress was also less prevalent in medical students who were more involved in organizing activities and medical volunteering as compared to those who were not (P = 0.039). Being involved in sports was also significantly associated with lower stress (high stress was prevalent in only 39.4% of medical students who did sports as compared to 54.7% who did not). See Table 4.

**Table 4:** Proportion of medical students with high stress and burnout scores in each of the domains with socio-demographic characteristics and involvement in extracurricular activities EE: emotional exhaustion; Cyn, cynicism; AE: academic efficacy Statistical analysis was performed using the chi-square test of independence. High burnout out was defined as having high EE (>14 points), high cynicism (>6 points), and low AE (<22 points).

		High EE			High Cyn			Low AE			High burnout			High stress		
		%		p	%		p	%		p	%		p	%		p
Gender	Female	87.9%	<0.001		64.4%	0.683		56.8%	0.499		41.7%	0.302		56.8%	0.137	
	Male	71.9%			66.5%			53.1%			36.2%			48.7%		
Age	18–24	78.0%	0.732		65.4%	0.585		53.9%	0.415		38.3%	0.942		52.7%	0.15	
	>25	75.0%			70.8%			62.5%			37.5%			37.5%		
Marital status	Single	77.0%	0.081		65.3%	0.386		53.9%	0.277		37.9%	0.548		51.9%	0.684	
	Married	100.0%			76.9%			69.2%			46.2%			46.2%		
Living arrangement	Parents/relatives	78.7%	0.081		65.9%	0.831		54.8%	0.662		39.2%	0.123		53.3%	0.018	
	Other	63.6%			63.6%			50.0%			22.7%			27.3%		
Academic year	1st year	83.1%	0.179		62.7%	0.066		54.2%	0.097		39.8%	0.119		61.4%	0.881	
	2nd year	78.6%			60.2%			52.4%			33.0%			39.8%		
	3rd year	75.0%			68.1%			43.1%			30.6%			52.8%		
	4th year	73.6%			69.8%			60.4%			45.3%			60.4%		
	5th year	75.6%			75.6%			71.1%			51.1%			48.9%		
GPA	2 - 2.74	92.9%	0.022		100.0%	<0.001		92.9%	<0.001		85.7%	<0.001		21.4%	0.01	
	2.75 - 3.74	82.8%			73.6%			63.2%			49.4%			65.5%		
	3.75 - 4.49	77.8%			68.6%			57.5%			39.2%			57.5%		
	4.5 - 5	71.6%			50.0%			37.3%			20.6%			35.3%		
Have you ever failed any course/block in college?	No	75.0%	0.041		60.9%	0.002		50.0%	0.006		31.3%	<0.001		50.4%	0.434	
	Yes	85.0%			78.0%			66.0%			56.0%			55.0%		
Do you smoke?	No	75.5%	0.052		64.4%	0.314		53.6%	0.521		36.7%	0.268		50.4%	0.345	
	Yes	85.9%			70.5%			57.7%			43.6%			56.4%		
Do you have any physician among first-degree family members?	No	77.2%	0.645		67.3%	0.318		58.3%	0.024		39.8%	0.339		53.5%	0.268	
	Yes	79.4%			61.8%			45.1%			34.3%			47.1%		
Extracurricular activities involvement (None)	No	70.2%	<0.001		63.5%	0.375		50.8%	0.158		33.1%	0.046		43.6%	0.002	
	Yes	85.7%			68.0%			58.3%			43.4%			60.0%		
(Student activity club)	No	79.7%	0.079		65.2%	0.642		56.9%	0.056		39.3%	0.367		54.5%	0.027	
	Yes	69.7%			68.2%			43.9%			33.3%			39.4%		
(Organizing activities and medical volunteering)	No	78.9%	0.079		65.1%	0.565		55.9%	0.191		38.2%	0.967		53.9%	0.039	
	Yes	71.2%			69.2%			46.2%			38.5%			38.5%		
(Research activity)	No	82.2%	<0.001		66.7%	0.404		55.6%	0.367		39.1%	0.456		53.5%	0.117	
	Yes	55.9%			61.0%			49.2%			33.9%			42.4%		
(Sports activity)	No	79.3%	0.175		67.0%	0.305		56.8%	0.075		40.7%	0.052		54.7%	0.021	
	Yes	71.8%			60.6%			45.1%			28.2%			39.4%		
(Video gaming)	No	78.7%	0.244		67.1%	0.114		53.9%	0.522		39.2%	0.263		52.0%	0.696	
	Yes	70.3%			54.1%			59.5%			29.7%			48.6%		
Statistical analysis was performed using the chi-square test of independence. High burnout out was defined as having high EE (> 14 points), high cynicism (> 6 points), and low AA (< 22 points).																

Binary logistic regression analysis shows that only a lower GPA was significantly associated with higher burnout rates (OR = 0.581, p 0.004, 95% CI= 0.400 to 0.843). None of the other variables was significantly associated with the odds of high burnout at the 0.05 significance level (Table 5).

**Table 5:** Binary logistic regression analysis results (R2 = 15.8) GPA: grade point average

	p	OR	95% CI	
			Lower	Upper
Gender (Male)	.375	.778	.446	1.355
Age (> 25)	.380	.594	.186	1.900
Marital status (Married)	.487	1.666	.395	7.021
Living arrangement (Parents)	.130	2.498	.764	8.167
Academic year	.232	1.142	.919	1.420
GPA	.004	.581	.400	.843
Failing a course or block (Yes)	.251	1.461	.765	2.792
Smoker (Yes)	.582	1.176	.661	2.093
Physician among first-degree family members? (Yes)	.690	.898	.530	1.523
None (Yes)	.096	2.026	.881	4.656
Student activity club (Yes)	.352	1.456	.660	3.209
Other (Yes)	.098	1.915	.887	4.134
Organizing activities and medical volunteering (Yes)	.501	1.306	.600	2.843
Research (Yes)	.872	.939	.439	2.008
Physical exercise (Yes)	.823	.919	.439	1.926
Video gaming (Yes)	.778	.881	.365	2.125
GPA and academic years were used as continuous variables				

Logistic regression results indicate that extracurricular activity was associated with stress. The odds of stress among medical students who do not have extracurricular activities is 89.3% higher than the odds in medical students who have an extracurricular activity (OR 1.893, P = 0.004, 95% CI=1.22 to 2.918). GPA was also associated with stress (OR = 0.737, P = 0.0.23, 95% CI=0.566 to 0.959). The odds of stress decreased with a higher GPA (26.3% decrease in the odds of stress with an increase in the GPA to a higher category) (Table 6).

**Table 6:** Multivariate logistic regression to assess independent predictors of stress GPA: grade point average

	OR	P	Lower 95% CI	Lower 95% CI
GPA	0.737	0.023	0.566	0.959
Extracurricular activities (None)	1.893	0.004	1.228	2.918

## Discussion

This study is one of the first attempts to assess the magnitude of stress and burnout among medical students in Saudi Arabia, and it is the first to analyze the role of extracurricular engagement and academic performance (GPA) in stress and burnout. The results of this study disclose a stress level of 51.7% and a rate of burnout was 38.2%, expressing high Exhaustion and high Cynicism scores. The literature reports varying rates of burnout among similar samples ranging between 49.6% and 76.8% [12-13]. Also, abundant studies on a vast range of health-related specialties note rates of burnout between 25% and 60% [14-15]. On the other hand, numerous studies note lower burnout levels ranging between 10.3% and 45% [16-18]. The findings of this research did not match the previous study done by Almalki et al. which reported alarming findings and reveal a high burnout level (67%) among medical students [19]. Several factors contribute to these varieties of results. Different underlying causes of burnout, requirements vary in each setting and using a different instrument since there are multiple instruments to evaluate burnout. Further, the criteria used in some studies (two-dimensional characterization) were less strict than the criteria in our study (three-dimensional characterization). In our study, burnout was present in 57.3% of the students when burnout was assessed as a two-dimensional scale and in 38.2% when assessed as a three-dimensional scale. We adopted the criteria (three-dimensional characterization) recommended by the researcher who developed the assessment instrument for burnout syndrome [3].

The rate of stress in our sample is much higher than others in the literature. A similar assessment of stress done in India and Turkey measured the stress risk in undergraduate medical students to be 28.4% and 25.6%, respectively, which is in contrast to our study 51.7% [20-21]. The high levels found in our study could be associated with a number of factors. One of which is the English language since the official school language in Saudi Arabia is the Arabic language. However, in our medical school, the student should score at least 5.5 in the International English Language Testing System (IELTS) or equivalent tests like the Test of English as a Foreign Language (TOEFL) to join the college. Moreover, the high level of competition among Saudi students due to the high number of graduates each year is also associated.

Our results agree with a study in Manchester, showing that students of the first year and fourth year had higher stress and burnout rates than those of other years [22]. Similar results also found in Lebanon were stress to be higher among first-year medical students (78.8%) [7]. A study in Nepal suggests that students gradually adapted to the new living environment, which can explain why always second-year medical students have a lower stress and burnout level than first-year students [7,22-23]. In addition, first-year medical students put under additional stress as well as to the normal, everyday stress, including workload, lack of time, new subject to be learned, and frequent examinations in a competitive environment. Also, a study from Pakistan suggests that the sources of stress for first-year medical students were found to be the course and curricular content, concern regarding a future, teacher's attitude, and possible career change [24]. Nevertheless, the fourth year is the first clinical year with just two years to graduation; students should build extraordinary resumes, publish research, and present at and attend conferences. On the other hand, some studies found that fourth and fifth-year medical students had the greatest level of stress as compared to junior medical students [17,25-26].

The current study showed that females have higher levels of burnout and stress. The finding is supported in the literature with studies reporting that women suffered burnout and stress more than men [7,17]. The higher female burnout and stress result in the current study could be associated with higher competition and a small number of acceptances every year in comparison to males. A study suggested that females are more likely to perceive challenging events as stressful as compared to men and some reported that female medical students had high-stress effects from contact with patients and autopsy more frequently than male students [18,27]. Yet, other studies imply opposite results where male students were at higher risk of burnout and stress than females [19,28-29].

Our study found significantly greater burnout and stress among low GPA students. The failure of blocks or courses in college serve as an additional stressor on the student, as they need to pass the subject to be with their colleague and finish the year; also GPA plays a major role in their future careers. Our results showed that stress and burnout were more prevalent in students who lived with their parents or relatives as compared to those who did not. This finding is at variance with another study that reports those living with their families to be suffering from lower burnout and stress, as only 20% of students living with the family had a high level of stress as compared with 41% living away from home [30].

An interesting finding in our results shows that high academic efficiency was more prevalent in individuals who have a first-degree relative working as a physician. This can be due to the high level of knowledge that the relative passes or the early exposure that the student will have. In other studies, students who have a physician among family members show significantly high burnout with high emotional exhaustion and high cynicism [17,19]. Also, another study stated that physicians’ parents are the second-highest source of stress for medical students [23].

Limitations

The study had several limitations, which should be considered in future research. The study was conducted in only one university despite a good response rate; further research should use a national approach. Another limitation of our study may be that of a non-response bias. This college only recently started admitting female students and, at the time of this study, had female students up to the third academic year only. Having a cross-sectional design is another limitation longitudinal studies are recommended, to confirm the relation.

## Conclusions

Our study shows high burnout rates among medical students. Low GPA students in this study showed a higher overall burnout rate. Stress was high in our study participants and was higher in students with a low GPA and in students who are not involved in any extracurricular activities. This study illustrates higher stress and burnout levels in first and fourth-year medical students. Academic counseling programs should be established especially for first and fourth-year students, to help them adapt to the new setting.
